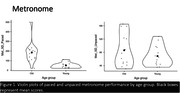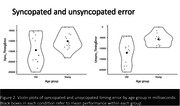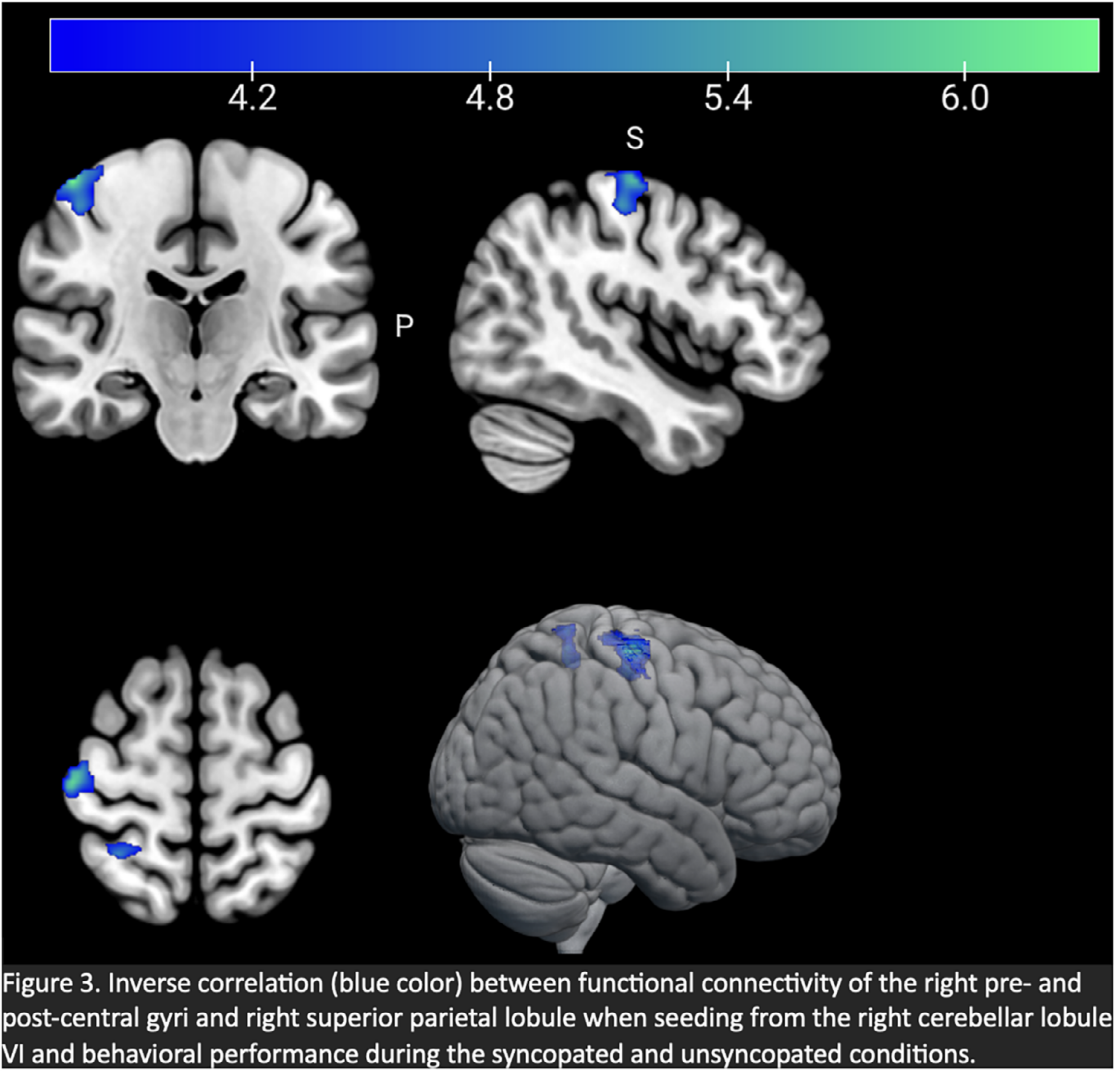# Aging, musical rhythm, and functional connectivity in the brain

**DOI:** 10.1002/alz.085605

**Published:** 2025-01-09

**Authors:** Aaron Colverson, Yu Chen, John B. Williamson, Ronald A. Cohen, Katherine P. Rankin, UCSF, Memory and Aging Center

**Affiliations:** ^1^ University of California, San Francisco, CA USA; ^2^ University of California San Francisco, San Francisco, CA USA; ^3^ University of Florida, Gainesville, FL USA; ^4^ Memory and Aging Center, UCSF Weill Institute for Neurosciences, University of California, San Francisco, San Francisco, CA USA; ^5^ Department of Neurology, Memory and Aging Center, University of California San Francisco, San Francisco, CA USA; ^6^ Global Brain Health Institute (GBHI), University of California San Francisco, San Francisco, CA USA

## Abstract

**Background:**

Aging associates with decreased functional connectivity between brain regions linked to musical rhythm perception. Producing rhythmic music may result in strengthened functional connectivity of these regions, but more evidence is needed to support intervention design. Currently, few studies directly contrast younger and older adults’ rhythmic music performance to understand brain‐behavior relationships. We used task‐based functional magnetic resonance imaging to examine aging‐related differences in connectivity among brain regions associated with perceiving and performing increasingly complex musical rhythms.

**Method:**

Participants (younger = 14, ages 18‐35; older = 14, ages 55‐79) completed one imaging run, sequentially tapping along to simple (paced and unpaced metronome, and unsyncopated) and complex (syncopated) musical rhythms on a response box. Simple rhythms maintained metric regularity, whereas complex rhythms deviated from the main pulse. Behavioral performance measures included overall timing error for the unsyncopated and syncopated conditions, and averaged standard deviation from mean error for paced and unpaced metronome. We ran seed‐to‐voxel analyses of known brain correlates (cortical and subcortical) of musical rhythm, contrasting effects of rhythm performance from each seed to examine functional connectivity.

**Result:**

Younger adults performed better than older during simple (paced metronome (F(1,26) = 20, p<0.001) and unsyncopated (F(1,26) = 13.2, p = 0.001)) rhythms, with a similar nonsignificant trend for complex rhythms (syncopated: F(1,26) = 3.77, p = 0.063). Seeding from the right cerebellar lobule VI, contrasting difference in performance during syncopated and unsyncopated blocks, a significant negative correlation was found with right pre‐ and post‐central gyri and right superior parietal lobule (cluster‐level, FDR corrected p<0.05, voxel threshold p<0.001). Applying the same contrasts while seeding from the left putamen yielded a significant negative correlation with the right cerebellum Crus I&II.

**Conclusion:**

Older adults performed simple musical rhythms less accurately than younger, and trended toward poorer accuracy in performing complex rhythms. During perception and performance of more complex musical rhythms, greater activation was found in circuits connecting the cerebellum (pattern abstraction) to parietal (timing representation) and basal ganglia (discrepancy monitoring) regions. Though we found no age difference, larger studies may be better powered to discriminate age‐related neural patterns. Studies investigating how music‐making interventions strengthen these circuits may provide avenues for improving cognition in aging adults and possibly individuals with neurodegenerative disease.